# Annotations

**Published:** 1890-02-01

**Authors:** 


### Annotations.

**Doctors and their Degrees.**

The outside public does not concern itself with the degrees of doctors, but employs practitioners on their individual merits, in exactly the same way as it employs bricklayers, tailors, carriage-builders, lawyers, and so on. This is a practice as intelligible as it is sound. When we enter within the sacred enclosure of the medical profession itself a new method is at once seen to obtain. Men are then measured by rules that apply to boys and youths, or, in other words, they are judged, not by their merits as adults and mature practitioners of their art, but solely by the arbitrary and uncertain merit of their academical qualifications. It is as if a general should be selected to conduct a great campaign because he had obtained more marks at Woolwich or Sandhurst than any of his contemporaries. Nothing more is wanted than this to show the embryonic condition of the medical mind when dealing with things medical. No fact could more convincingly demonstrate the all-pervading want of faith felt by doctors in their own art than this resolute selection of men on academic grounds rather than on grounds of proved practical skill. If anyone were to propose to choose a soldier, an engineer, or even a maker of match boxes, on any such principles he would very properly be regarded as a candidate for Bedlam. This is a very old question, and one which no doctor who has passed beyond the intellectual status of a baby ever discusses if he can help it. Recently the town of Derby and its hospital managers compelled all who have opportunities and the well-being of the medical profession at heart, to enter a protest against their proceedings. Those gentlemen had, quite unwittingly, no doubt, lent themselves to the entirely disgraceful and wholly unjustifiable practice of "boycotting." They had pronounced a most momentous verdict on a subject which the vast majority of them can know nothing whatever about. Most of them, though outside the medical profession, had confidently assessed the value of different medical degrees. No doubt they will plead that they have acted under the guidance of a majority of their medical staff. But if they enquire carefully they will find that they have followed the lead of doctors who have played the parts of plaintiff, judge, and jury in their own cause. Those gentlemen, knowing something of the work involved in obtaining their own degrees, but necessarily not knowing anything whatever, except by hearsay, of the work involved in obtaining other degrees, have yet, with a modesty as rare as it is excessive, decided that their own degrees deserve hospital appointments whilst some of their neighbours do not. We have said that to select physicians and surgeons for hospital appointments on the ground of their academic degrees, instead of on that of their proved practical skill, is in itself entirely absurd. But even if it were not, can anything be more truly ridiculous than to permit one medical man to assess the merits of his own degree as compared with those of others? Of course he will say his own is the best, exactly as he will say his own nose is the handsomest, his own skill the highest, his own merits the most conspicuous in any and every department. If the Derby managers had had no light to guide them, they might have been excused. But they had the authoritative guidance of the General Medical Council of Great Britain and Ireland. That council has declared that certain qualifications entitle the holders thereof to practise medicine, surgery, and midwifery among all classes of Her Majesty's subjects and in every part of the United Kingdom. The Derby managers, without a shadow of statesmanlike sense or reason, and apparently at the bidding of the holders of one or two particular qualifications—qualifications that are in no single particular superior to or even different from those whose holders are excluded—had by regulation proclaimed a solemn "boycott" against men whom the General Medical Council thus authorises to practise every branch of medicine among all ranks of the Queen's subjects. Such a regulation is absurd in itself, and it is iniquitous and injurious in its effects upon all the duly qualified men whom it excludes. It sets a brand upon them. It lowers their prestige with their own patients and the public. It exalts the included ones unjustly. The whole medical profession owes it to itself and to each of its members to protest against, to denounce, and to terminate at once such a gross injustice. We have written strongly because we feel strongly that mere unprofessional ignorance ought not to be utilised by a few medical men for their own advantage. We understand that more than one or two of the present medical staff at Derby practise pharmacy, that is, dispense their own medicine and also take midwifery cases. If this be so how can they with any grace vote for the exclusion from the staff of any medical man on the register. For our part we would advise the new committee just elected at Derby to call in an unprejudiced and experienced expert free from local prejudices to assist them in the present deadlock, whilst they determine to prohibit every physician holding office at the infirmary from practising pharmacy. It is, as we have said, not a question of degrees, but of competency and standing. No hospital physician must be allowed to dispense medicines, neither may any registered practitioner be appointed a hospital surgeon unless he has previously had considerable practice in operative surgery. Merit, not degrees, is the only true test for hospital appointments.

**Lotteries and Hospital Funds.**

A Constant Reader writes: "The article in last week's issue of The Hospital, under the heading of 'Fallow Crops,' has led me to consider whether or not some scheme could not be devised for obtaining subscriptions to hospital funds which, whilst possessing some of the attractions of a lottery scheme, would at the same time be free from its objections, and, consequently, not liable to be interfered with as a violation of the law. It has occurred to me that a scheme something like the following might be worthy of consideration: Let there be drawings at intervals of, say, every three months, in which purchasers of tickets costing 10s., 5s., 2s. 6d., and 1s. each shall have the right to participate. The prizes to be drawn for being the privileges, in the shape of patronage and otherwise, attaching to life governorships, ten years' subscribers, five years' subscribers, and annual subscribers respectively. The number of prizes to be drawn for being, of course, limited according to the amount produced from the sale of each class of tickets. This, I venture to suggest, would be a great inducement to a large number of subscribers, would be very inexpensive in carrying out, and, as no portion of the funds would be returnable in the shape of prizes, the whole amount subscribed would be a net profit to the institution, and, as it would be simply an extension of the co-operative principle to the purchase of the privileges to be gained, could hardly be considered a lottery within the meaning of the Lotteries Acts. The holders of lucky tickets would also add a large contingent to the ranks of those taking an active interest in hospital work." The proposal our correspondent makes is doubtless an innocent one, and no harm could come of trying to give effect to it, but we fear that the prizes are not attractive enough to make it likely that the result will be of any practical value. If a lottery is to be successful, the prizes must be such as will arouse the acquisitive propensity of the multitude. Will the prospect of becoming governors or members of hospitals do this? We doubt it. Excepting for its illegality, the proposal we lately commented upon—to raffle a cash prize—was the essence of worldly wisdom. Given a golden lever and a legal fulcrum and it would not be difficult to raise half a million pounds annually to the level of our hospitals' treasuries.

